# *Streptococcus pneumoniae*: ‘captain of the men of death’ and financial burden

**DOI:** 10.1099/mic.0.001275

**Published:** 2022-12-02

**Authors:** Hasan Yesilkaya, Marco R. Oggioni, Peter W. Andrew

**Affiliations:** ^1^​ Department of Respiratory Sciences, University of Leicester, Leicester LE1 7RH, UK; ^2^​ Department of Genetics, University of Leicester, Leicester LE1 7RH, UK; ^3^​ Dipartimento di Farmacia e Biotecnologie, University of Bologna, Bologna, Italy

**Keywords:** *Streptococcus pneumoniae*, pneumococcal infections, virulence, genome

## Abstract

*
Streptococcus pneumoniae* may inhabit the upper respiratory tract of humans without causing harm but it also causes diseases with high morbidity and mortality. It has excellent adaptive capabilities thanks to its ability to shuffle its genetic content by acquiring and incorporating DNA from other bacteria and is highly competent for genetic transformation. Sugar sensing, cleavage and transport ensure its fitness and survival in the host, and intracellular survival in macrophages has been linked to virulence. The polysaccharide capsule and toxin pneumolysin are the most important virulence determinants. Polysaccharide-based vaccines provide protection against the serotypes represented in vaccine formulations.

## Taxonomy

Phylum: *Firmicutes *; Class: *Bacilli *; Order: *Lactobacillales *; Family: *Streptococcaceae *; Genus: *Streptococcus *; Species: *Streptococcus pneumoniae *. Lancefield serological grouping of streptococci based on the presence of polysaccharide and teichoic acid antigens in the bacterial cell wall designates *S. pneumoniae* as ungroupable because pneumococcal antigen extracts do not react with streptococcal group antisera.

## Properties

*S. pneumoniae* is a Gram-positive, non-motile, facultative anaerobe with more than 100 known serotypes, each representing a chemically and genetically unique capsule type. Lancet- or spherical-shaped pneumococcal cells appear in pairs or short chains. On blood agar, it forms α-haemolytic greenish-coloured colonies. It can grow at 28–42 °C. The pneumococcus does not respire: fermentation is the only mode of energy generation. Biochemical tests to differentiate the pneumococcus from other streptococci include catalase and oxidase negativity, lysis by bile salts and sensitivity to ethylhydrocupreine (optochin). Supplementation of medium with catalase enhances growth by eliminating the high level of endogenously produced H_2_O_2_.

## Genome

The pneumococcus has a circular AT-rich (~60 %) genome of approximately 2.1–2.3 Mb, encoding approximately 2000–2200 genes [1]. The genome contains a high number of genes coding for glycosidases, sugar metabolic pathways, sugar transporters and signalling pathways required for nutrient detection, such as canonical two-component regulatory- and quorum-sensing systems, showing the integral role of sugar metabolism in pneumococcal biology. There is a high level of genome plasticity among isolates. Analysis of 13 454 genomes showed that 1276 genes were found in more than 95 % of genomes, representing the core genome. An additional 1957 genes were shared by many genomes (≥15 to <95 %), whilst 24 219 genes were shared by a small subset of genomes in the pangenome [2]. *S. pneumoniae* has an open pan-genome unlike other bacteria such as *Salmonella enterica* or *Mycobacterium tuberculosis* which have a closed pangenome [3]. Recombination rather than spontaneous mutation is the key evolutionary force for the pneumococcal genome [4]. Recombination is driven partly by transposable elements (~5 % coding capacity) and by the ability to uptake and incorporate naked DNA from the surrounding environment into the native genome, also known as natural competence. Microbial genome-wide association studies have confirmed increased recombination in loci under selective pressure, for example in the penicillin-binding proteins and the capsular polysaccharide locus.

## Phylogeny

Based on 16S rRNA phylogenetic analysis the pneumococcus is a member of the Mitis clade within the non-pyogenic (not β-haemolytic) viridians group streptococci (VGS), which also include the Anginosus, Salivarius, Sanguinis and Mutans clades. The Mitis sub-group includes pathogenic, *S. pneumoniae *, and non-pathogenic species, such as *S. pseudopneumoniae *, *S. mitis* and *S. infantis *, residing in the oropharynx and nasopharynx [5]. The Mitis group was suggested to have evolved from a pneumococcus-like bacterium likely to have been pathogenic for the common immediate ancestor of hominoids approximately 6–7 million years ago. Pneumococcal virulence potential has been attributed to the flexible gene pool. The pneumococcal strains can be differentiated from other viridans groups and phylogenetically grouped by multilocus sequence typing, which relies on sequence analysis of seven housekeeping genes or whole genome sequencing, which is a highly accurate and sensitive analysis tool [6].

## Key features and discoveries

Branded as ‘captain of the men of death’ by William Osler in 1918, *S. pneumoniae* was characterized initially by Louis Pasteur in 1881. Despite being considered an extracellular bacterium, recently its survival within liver and spleen macrophages has been linked to the onset of invasive pneumococcal disease [7].

The pneumococcus spreads through respiratory droplets. It colonizes the upper respiratory tract as a commensal. The rate of colonization is higher among infants than among adults and is greater in Africa than in Europe. The invasive phenotype ensues local spread via aspiration or direct seeding into the bloodstream. The pneumococcus is the leading cause of bacterial pneumonia, killing 1.5 million people of all ages, and a major agent of meningitis and septicaemia as well as other debilitating diseases such as otitis media, septic arthritis, keratitis and sinusitis [8]. Poor living conditions, physiological stress, smoking and asplenia are predisposing factors for pneumococcal infections.

Several proteins and physical structures contribute to pneumococcal virulence by promoting nutrient acquisition, mediating colonization and transmission, modulating translocation into tissues, and shielding the microbe against the host immune system. The notable virulence determinants include a polysaccharide capsule, the cytolytic toxin pneumolysin and glycosidases [8]. Located in the outermost layer of the cell envelope, the capsule prevents pneumococcal agglutination by mucin, modulates adhesion and transmission, and protects pneumococcal cells through inhibition of complement deposition. The microbe can switch its capsule. While their geographical distribution is uneven, all serotypes retain their virulence potential, and different serotypes vary in their ability to colonize as well as cause invasive diseases. Pneumolysin lyses host cells by forming pores. It inhibits ciliary beat and complement activation, and modulates immune cell function by affecting cytokine and chemokine production [9, 10]. The pneumococcal interaction with host glycans is critical for its colonization and invasive capabilities, and *in vivo* nutrition. Pneumococcal glycosidases, such as galactosidases and neuraminidases, combine to sequentially cleave complex host glycans. In doing so, they expose host receptors for adhesion and also provide sugars for energy generation.

The World Health Organization designated *S. pneumoniae* as one of 12 priority pathogens, requiring effective anti-infectives due to the increasing trend of antibiotic resistance. Capsular polysaccharide and 13-, 15- and 20-valent polysaccharide–protein conjugate vaccines are available, which protect against colonization and invasive pneumococcal diseases but only by those serotypes represented in the vaccine formulation.

## Open questions

1. What are the *in vivo* nutrient requirements of *S. pneumoniae *?
2. What physical and chemical signals trigger pneumococcal switch from asymptomatic carriage to invasive phenotype, and what are the pneumococcal changes in response to the signals?
3. By which cell types does the pneumococcus translocate from nasopharynx to other host tissues?
4. Is a pan-serotype vaccine possible?
5. What are the microbial features that determine infection outcomes in the host?
